# Tips and tricks for gut microbiota investigation using scanning electron microscopy (SEM): going from sample preparation to imaging and landscape analysis

**DOI:** 10.1080/19490976.2025.2512016

**Published:** 2025-06-09

**Authors:** Meriem Boukili, Omar Zmerli, Florence Fenollar, Sara Bellali, Jacques Bou Khalil

**Affiliations:** a Aix Marseille Univ, MEPHI, Marseille, France; b IHU Méditerranée Infection, Marseille, France; c Aix Marseille Univ, APHM, SSA, RITMES, Marseille, France; d Aix Marseille Univ, APHM, MEPHI, Marseille, France

**Keywords:** Gut microbiota, scanning electron microscopy, dysbiosis, *Clostridioides difficile* infection

## Abstract

The Gut Microbiota (GM) remains a complex microbial ecosystem with many unknown facets despite significant technologic advancement. This study introduces a novel rapid technique using tabletop scanning electron microscopy (SEM) for investigating GM composition, focusing on *Clostridioides difficile* infection (CDI) as a representative model for dysbiosis-related diseases. Six stool sample preparation protocols were tested on 40 stool samples to develop an optimized SEM protocol. Protocol stability was evaluated after four-month storage. The optimized protocol produced high-resolution micrographs while maintaining sample integrity over time. SEM investigation of GM was done by analyzing ten stool samples (5-control and 5-*C. difficile* groups), imaged at low and high magnifications. Object detection analysis generated a SEM-based GM components database helping describe and compare microbial diversity variation between the groups. CDI group revealed a reduction in microbial diversity, compared to the controls. Epithelial and red blood cells were more prevalent in CDI group. Statistical analyses of objects proved clear clustering of samples into CDI and control groups. This study pioneers the proof-of-concept for using tabletop SEM to investigate GM components in a dysbiosis-related disease model. This concept emerges as a complementary technique capable of providing deeper insight to describe GM components previously elusive with other methods.

## Introduction

The Gut Microbiota (GM) is a highly complex and dynamic microbial ecosystem harboring various microorganisms; predominantly bacteria, alongside fungi, archaea, and viruses.^1–4^ Despite its diversity, the GM exists in a functional equilibrium, known as “*eubiosis”*, which contributes to a range of biological processes, including digestion, modulation of the immune system,^5^ and neurological well-being through its bidirectional communication with the central nervous system via the gut-brain axis.^6,7^ However, this symbiotic balance is prone to disruption by various exogenous and endogenous factors leading to gut “*dysbiosis”*: a state characterized by an imbalance in microbial composition, reduced diversity, and overgrowth of pathogenic species. Gut dysbiosis has been associated with multiple diseases including gastrointestinal disorders such as inflammatory bowel disease (IBD),^8^ metabolic disorders like obesity and diabetes,^9,10^ and neuropsychiatric disorders including anxiety, depression, and cognitive decline.^11^ Notably, *Clostridioides difficile* infection (CDI) is among the most studied dysbiosis-related diseases. This worldwide infection represents a leading cause of healthcare-associated infections (HAIs) in the United States, with an estimated incidence in 2022 of 116.1 cases per 100,000.^12^ It is principally triggered by antibiotic therapy, which promotes *C. difficile* spore germination, bacterial overgrowth, and toxin production including toxins A (TcdA) and toxin B (TcdB).^13^ Toxin A disrupts the intestinal epithelial barrier and attracts neutrophils, while toxin B disrupts the host cell cytoskeleton, leading to cell death.^13^ Both toxins, encoded by the pathogenicity locus in the *C. difficile* genome, causing a pro-inflammatory response in the intestinal epithelium,^13,14^ leading to clinical manifestations ranging from mild diarrhea to severe pseudomembranous colitis and death.^15^ Despite the high efficacy of current treatments, including antibiotics and fecal microbiota transplantation (FMT) for recurrent cases, CDI remains a significant clinical challenge due to high recurrence rates.^16,17^ The mechanisms driving recurrence are complex and involve many variables including spore persistence, the extent of microbiome restoration and the role of the host’s immune system.^18–20^ Furthermore, the absence of efficient methods for post-treatment monitoring of GM changes is a major challenge for identifying the root cause of disease recurrence. Such approaches could enable clinicians to identify therapeutic targets such as specifically lacking GM components or gain access to a more tangible characterization of GM dynamics.

Given the established role of GM composition in maintaining homeostasis and its implication in various disease states, such as CDI, a multitude of tools have emerged with the common aim of investigating GM components, which can be referred to as a landscape of several components including microorganisms and cells.^21,22^ The development of multi-omics approaches allowed a better, yet incomplete, characterization of the GM composition, functions, and dynamics. Sequencing-based methods such as metagenomics,^21^ and culture based-techniques, including culturomics,^22^ are among the leading omics approaches used to date. The culturomics approach is limited to the detection of live bacteria that are culturable, by means of a complex technique involving a multitude of conditions and prolonged incubation time.^23^ Sequencing-based techniques such as metagenomics can provide a quasi-complete description of the sample’s microbial content based on the detection of nucleic acids. However, despite the quantity of information this approach provides, this form of detection does not always reflect the actual presence of the microorganisms in the sample due to sensitivity/specificity challenges. Therefore, a significant knowledge gap remains present limiting our ability to accurately describe GM composition.^24–26^

In this context, this work introduces a novel proof-of-concept for a rapid tabletop Scanning Electron Microscopy (SEM)-based investigation of GM composition, using CDI as a dysbiosis-related disease model, aimed at presenting a complementary approach for existing methods, ultimately providing deeper insight into the GM landscape for a more complete and tangible analysis of the GM composition.

## Materials and methods

### Ethics statement and study design

**Figure 1.**
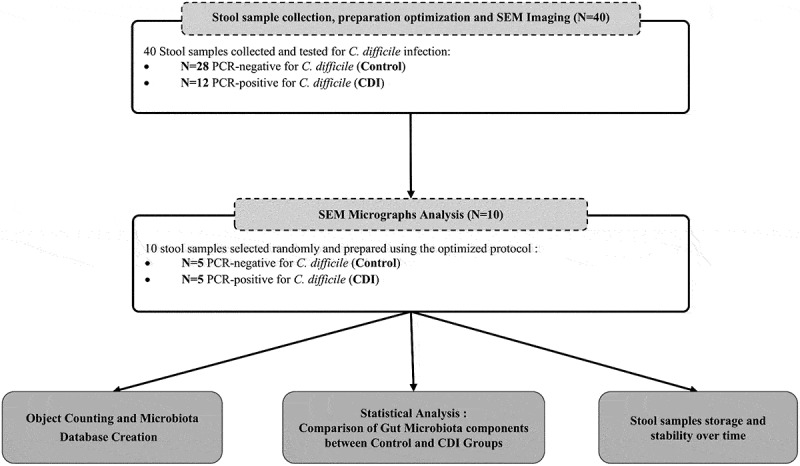
Overall study design flowchart.

This study included a total of 40 stool samples (Figure 1) collected as laboratory waste from the clinical microbiology laboratory at the *Institut Hospitalo Universitaire (IHU) Méditerranée Infection* in Marseille, France, between October 17, 2024, and January 31, 2025. The study received ethical approval (No. CSE24–19) from the Ethics and Scientific Committee of Assistance Publique – Hôpitaux de Marseille (APHM).

The collected stool samples were randomly selected from discarded samples from the clinical laboratory, including samples with various volume and consistency. These samples were originally tested for *Clostridioides difficile* infection (CDI) as part of the routine diagnostic pathway, using an in-house polymerase chain reaction (PCR) test. Samples were classified into two groups: control (PCR-negative for *C. difficile*) and CDI (PCR-positive for *C. difficile*).

### Stool sample preparation optimization

Several stool sample processing protocols were tested to establish an optimized sample preparation protocol that remains stable despite the diversity of stool properties like volume, texture, and contents. The optimal protocol was chosen to allow easy SEM observation, rapid sample preparation, and the preservation of the integrity of the sample’s components. Moreover, the choice of protocol rested on its ability to produce a homogenous deposition of the stool sample on a SEM-suitable support. These features were essential to guarantee an adequate and reproducible analysis of the diversity and richness of the stool sample.

**Figure 2.**
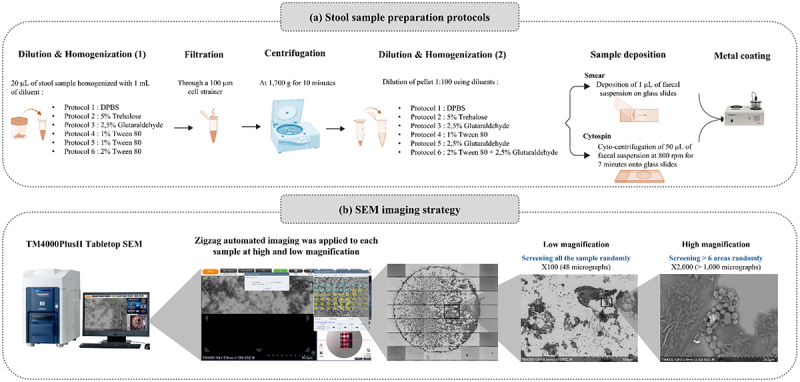
Overview of the workflow. (a) Optimizing stool sample preparation protocols. (b) Zigzag automated imaging SEM strategy at low and high magnifications.

Initially, the stool sample was processed by performing direct smearing and freeze-drying, however, these methods were abandoned due to result variability and long preparation times. Overall, six protocols were tested by varying the diluents used to process the sample in each step, as detailed in Figure 2a. First, a fixed 20 µL aliquot of stool was homogenized using 1 mL of a diluent. The homogenized suspension was then filtered through sterile 100 µm filters to remove food debris and centrifuged at 1,700 g for 10 minutes forming a pellet. This low centrifugation speed was selected in order to gently pellet the sample contents without disrupting their natural surface charge or inducing significant artificial aggregation, ultimately avoiding clumping and structural damage.^27^ The pellet was then resuspended/homogenized and diluted (1:100) in either the same diluent or with an adjusted diluent. Following the second homogenization step, two methods of sample deposition on glass slides were evaluated: (1) sample smearing, where 1 µL of the fecal suspension was manually spread on the slide, and (2) Cytospin deposition (Thermo Fisher Scientific, United States) of 50 µL of fecal suspension at 800 rpm for 7 minutes. Once the slides were completely dry, a thin layer of sputter-coating with platinum-palladium was applied using the MC1000 Ion Sputter Coater (Hitachi High-Tech, Japan) to reduce electron charging effect during SEM imaging (Figure 2a).

The choice of different diluents for each of the homogenization steps was done based on the physicochemical properties and known effects of these diluents on biological matter. A wide range of diluents including buffers, detergents, and fixative agents were tested for the initial sample homogenization step and the subsequent pellet homogenization step, including: Dulbecco’s phosphate-buffered saline (DPBS) (Life Technologies, United Kingdom), 5% trehalose (MP biomedicals LLC illkirch, France) diluted in distilled water, 1–2% tween 80 (Merck KGaA, Germany) diluted in Muller-Hinton broth (Millipore Sigma, Germany) and 2.5% glutaraldehyde (Electron Microscopy Sciences, United States) diluted in 0.1 M sodium cacodylate buffer (Thermo Fisher Scientific, United States). The pH of all diluents was adjusted to 7.3 ± 2 and all diluents were filtered through sterile 0.22 µm filters.

### SEM imaging strategy

The prepared slides were imaged using the automated image acquisition function (Zigzag) of the novel tabletop TM4000PlusII SEM (Hitachi High-Tech Corporation, Japan). The overall acquisition workflow was carefully designed to capture multiple representative areas of the sample. First, the entire slide was imaged at a low magnification (x100), yielding an average of 48 micrographs per slide, covering a surface area of 58 mm^2^. This provided an overview of the sample and allowed the visualization of large objects, with clear borders around the sample deposition zone. Second, to better visualize smaller objects, notably bacteria and yeast, multiple random areas within the sample deposition zone (average surface area 25 mm^2^) were imaged at high magnification (x2,000) generating 80–150 micrographs per area, yielding more than 1,000 micrographs per slide, covering an average surface area of 3 mm^2^. (Figure 2b). All micrographs were acquired at an acceleration voltage of 10 kV using the BSE (Backscattered Electron) detector. Acquisition settings are visible on each micrograph in the following format: Instrument, Accelerating Voltage, Working Distance, Magnification, and Detector.

### SEM analysis strategy: GM composition definition and object classification

As shown in the study design (Figure 1), 40 stool samples were processed using the optimized sample preparation protocol, followed by SEM image acquisition. In total, more than 40,000 micrographs were captured. A preliminary manual SEM-based GM composition analysis was conducted on 10 randomly selected samples: 5 from the CDI group and 5 from the control group. Object identification analysis was performed on 96 randomly selected micrographs per sample: 48 at low magnification (x100) and 48 at high magnification (x2,000). A manual count and annotation of objects based on morphological characteristics for each object, including shape, size, and grouping/distribution pattern was done using ImageJ Fiji software. This allowed the creation of a database containing all the measured variables for each object.

### Stool samples storage and stability over time using the optimized protocol

Following the optimization of the stool sample preparation protocol, a series of experiments to determine sample integrity preservation were done to ensure the stability and reproducibility of results obtained from stored samples. A four-month storage period was set as a target, and the remaining volume from the initial 40 processed stool samples was stored at + 4°C. After four months of storage, slide preparation and image acquisition steps were repeated for the same subset of 10 previously analyzed samples using the same acquisition conditions. The two sets of images (initial/stored) for the selected subset were then comparatively analyzed to evaluate changes in the morphological characteristics of identified objects over time (Figure 1). The stability of the commonly encountered microorganisms composing GM was evaluated by analyzing objective morphological parameters (surface area, circularity, width, and percentage of deformation) for bacilli, yeast and cocci. Yeasts were analyzed provided their well-described sensitivity to osmotic shock which could result from environmental changes.^28^ Bacilli and cocci representing the bacterial content of the GM were analyzed to describe their overall morphological integrity. All measurements were done using ImageJ Fiji software. A detailed description of this comparative analysis methodology is shown in Supplementary Figure S1.

### Statistical analysis

All statistical analyses were performed using RStudio version 4.4.2. To evaluate the relationship between identified object counts and sample groups, the counts were analyzed in a reduced-dimensional space by applying a Principal Component Analysis (PCA). Following PCA, k-means clustering was applied to group the samples into two distinct clusters based on their principal component scores. The quality of the clustering was evaluated using the Silhouette Score, Davies-Bouldin Index (DBI), and Calinski-Harabasz Index. A PCA biplot with confidence ellipses at a 0.95 level was generated to visualize the clusters and their dispersion. The FactoMineR, Factoextra, ggplot2, clusterSim, and corrplot packages were used for these analyses. Additionally, three alpha diversity indices – Shannon’s diversity index, Simpson’s diversity index, and Pielou’s Evenness index – were calculated using the vegan package and visualized as boxplots using the ggplot2 package. Quantitative results were reported as mean and standard deviation. Statistical differences in variable means between the two groups were assessed using the Mann – Whitney U test, with p-values less than 0.05 considered statistically significant. Visualization of results for object stability measurements was done using Prism software, version 8.0 (GraphPad Software, San Diego, CA).

## Results

### Sample preparation protocol optimization

By evaluating six different protocols for optimizing stool sample preparation, significant variations in final sample homogeneity and image quality were observed. The preliminary experiments testing both sample deposition methods revealed that sample smearing produced overall heterogenous distribution of the sample and reduced visibility of objects due to variable artifacts (data not shown). Therefore, this method was excluded from the sample deposition methods, retaining only Cytospin sample deposition, as it yielded rapid reproducible results with a homogenous sample distribution on glass slides.

**Figure 3.**
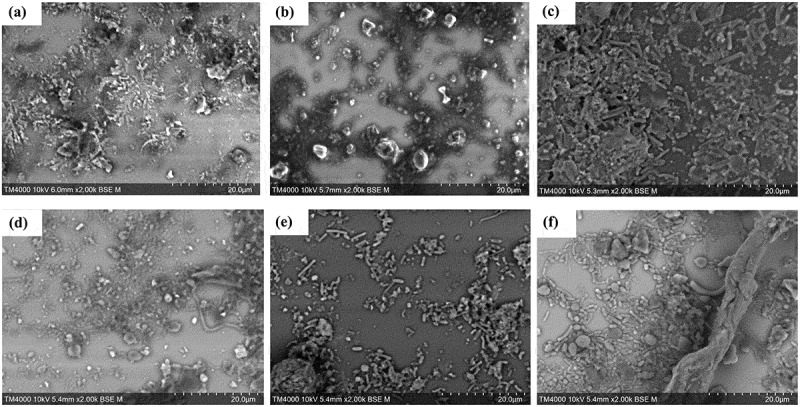
SEM micrographs of stool samples generated using the six tested protocols. (a) Protocol 1, (b) protocol 2, (c) protocol 3, (d) protocol 4, (e) protocol 5, and (f) optimized protocol 6.

Figure 3 demonstrates the results obtained for all tested protocols in the optimization process. Micrographs obtained with Protocol 1 exhibited predominant crystallization of the sample, likely due to the presence of salts, such as sodium chloride (NaCl), in the DPBS buffer (Figure 3a). Protocol 2 and 3, which included trehalose (5%) and glutaraldehyde (2.5%) as fixatives to protect bacterial cells from environmental stress, respectively, showed a significant aggregation of GM components, resulting in blurred micrographs (Figure 3b,c). Protocol 4, which used Tween 80 (1%) as a detergent, to reduce biofilm formation and facilitate the dispersion of sample components, allowed for an easier visualization of sample components, however, these remained unclear on SEM micrographs (Figure 3d,e). Protocol 5 consisted of adding glutaraldehyde (2.5%) followed by Tween 80 (1%), but the results remained similar to those obtained with Protocol 4. Finally, protocol 6 evaluated the use of Tween 80 (2%) and glutaraldehyde (2.5%), which significantly enhanced image clarity with minimal object aggregation. Therefore, protocol 6 was selected as the optimized protocol, which was applied to all samples. This optimized protocol allowed a clear visualization of the diverse morphologies/shapes of the objects comprising the GM (Figure 3f).

### GM component definition and object classification

**Figure 4.**
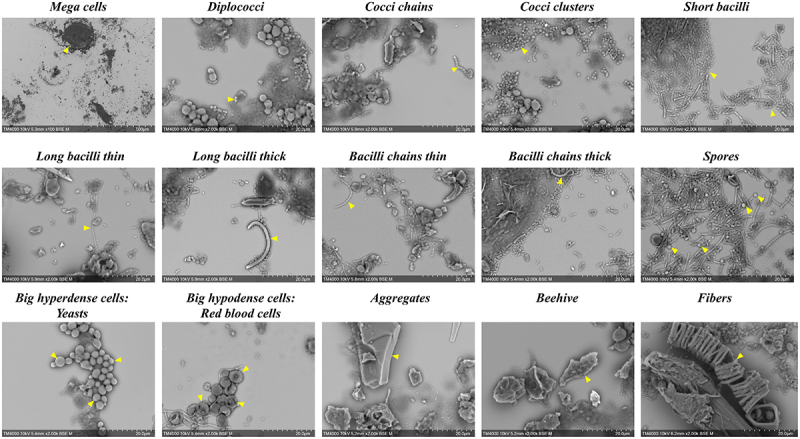
Presentation of the 15 objects detected in stool samples prepared using the optimized protocol.

Image analysis for the 10 randomly selected samples enabled the detection and identification of a total of 15 distinct objects as shown in Figure 4. At low magnification (x100), large objects exceeding 40 µm in their longest axis were annotated as “mega cells”, primarily corresponding to epithelial cells and other intestinal cells. At high magnification (x2,000), several forms of bacteria were annotated: diplococci, cocci chains, cocci clusters, short bacilli, long bacilli thin, long bacilli thick, bacilli chains thin and bacilli chains thick. Additionally, yeasts were identified and annotated as “big cells-hyperdense” due to their high electron density on SEM micrographs, in comparison to other components of similar size. Other cells of human origin such as red blood cells, were also observed and annotated as “big cells- hypodense” due to their lower electron density on SEM micrographs, especially in comparison to yeasts. Several other objects were also identified, including aggregates, spores, beehive-like structures, and fibers (Figure 4). However, the appearance of these objects was variable and sporadic, making their annotation difficult given the small number of samples. Therefore, they were excluded from this analysis.

**Figure 5.**
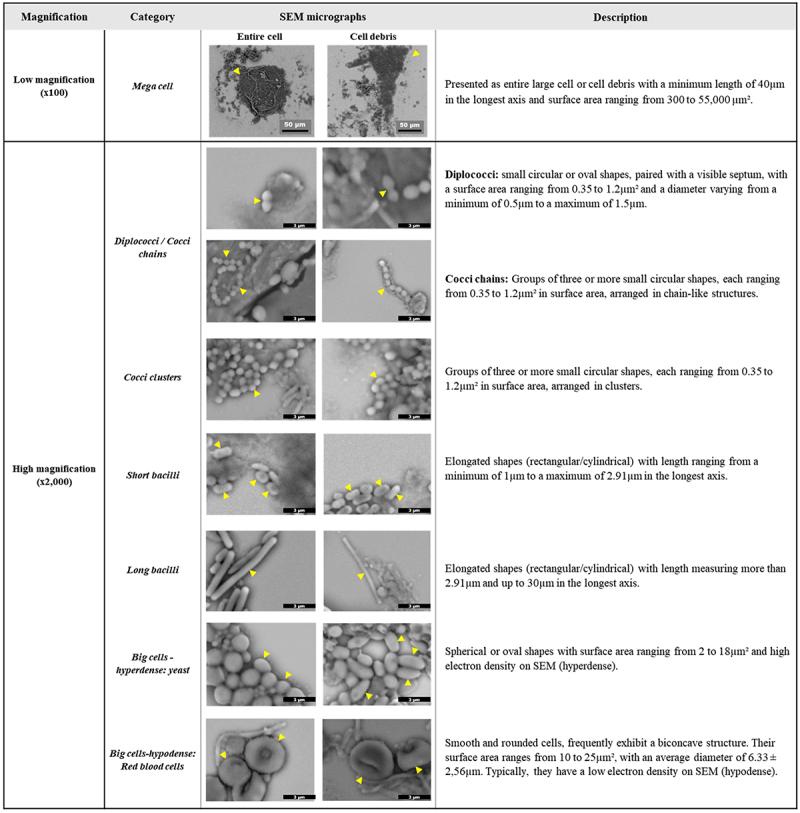
Detailed description of the seven objects observed on SEM micrographs.

Finally, seven primary objects were set as the targets for analysis since they were the most frequent across all samples, facilitating the manual annotation and the creation of an object database with quantitative and qualitative characteristics. A detailed morphological description of each of the analyzed objects is shown in Figure 5.

### Comparison of gut microbiota between control and CDI groups

#### GM composition analysis by group

**Figure 6.**
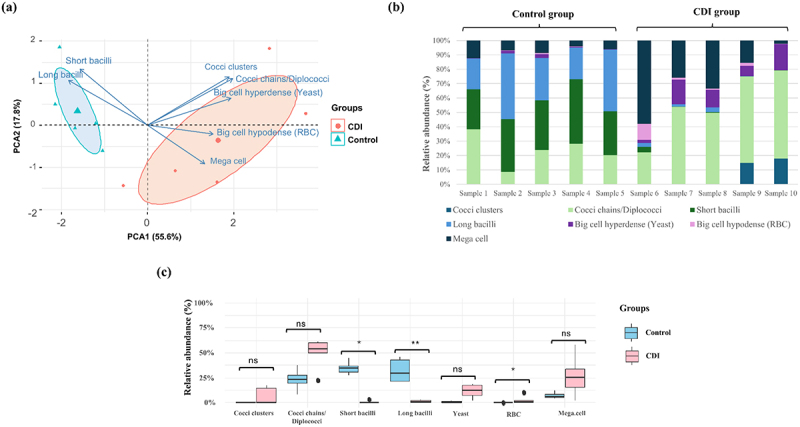
Gut microbiota composition analysis.

GM composition for each sample was determined based on the seven annotated objects in the database for both CDI (*n* = 5) and control (*n* = 5) groups (Figure 5). The PCA facilitated the observation of the distribution of objects among different samples in the two groups, providing their relative positions in the reduced dimensional space. Following PCA, k-means clustering was applied to group the samples into two clusters. The quality of the clustering was evaluated using several metrics, which provided evidence for the distinctiveness of the clusters. Specifically, the average silhouette width was 0.48, indicating moderate cohesion and separation of the clusters. The Calinski-Harabasz index was 13.06, suggesting good between-cluster variance relative to within-cluster variance. Additionally, the Davies-Bouldin index was 0.78, which is below 1, indicating well-separated clusters. This clustering allowed the identification of the contributing objects per sample group, highlighting differences in the gut microbiota (GM) components between the two groups (Figure 6a). Analysis of the relative abundance of objects for each sample revealed a high similarity of GM composition within the control group (Figure 6b). These objects were distributed relatively equally, with the absence of a predominant microbial shape dominating the population. As for the CDI group, the analysis showed a significant reduction of short bacilli (*p* = 0.011) and long bacilli (*p* = 0.007), a significant increase in red blood cells (*p* = 0.03), and a relative increase in yeast (*p* = 0.09) and mega cells (*p* = 0.54) (Figure 6c).

(a) Principal component analysis (PCA) biplot of GM composition in the Control and CDI groups. Points represent individual samples, arrows indicate object contributions in the reduced dimensions, and confidence ellipses (95%) highlight the clustering achieved through k-means clustering, demonstrating clear differentiation between groups. (b) Relative abundance of the predefined objects, illustrating GM composition in Control and CDI groups. (c) Bar-plot analysis demonstrated significant differences in gut microbiota objects marked by imbalance in CDI group. Error bars represent the standard deviation (SD).

#### Alpha diversity analysis

**Figure 7.**
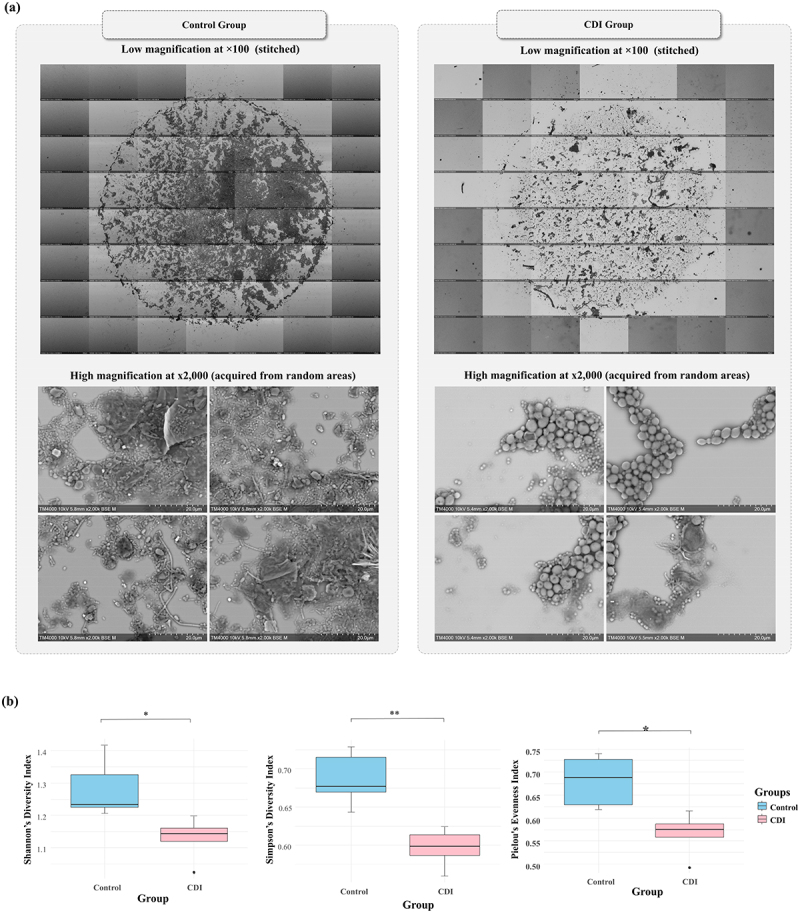
Comparison of gut microbiota diversity between control and CDI groups.

The SEM micrographs and corresponding α-diversity indices for detected objects showed a significant reduction in GM diversity within the CDI group compared to the control group (Figure 7). Stitched low magnification (x100) micrographs revealed a lower richness in the CDI group as compared to the control group. Similarly, high magnification micrographs (x2,000) revealed a lower microbial diversity in the CDI group, as compared to the control group, which was mainly characterized by the presence of diverse microbial objects, including cocci and bacilli with several sizes and distribution patterns (Figure 7a). These morphological findings were statistically verified with the Shannon’s diversity index revealing a significant decrease in GM richness (1.28 ± 0.09 in the control group vs. 1.13 ± 0.07 in the CDI group; *p* = 0.012), and the Simpson’s diversity index highlighting the overall disruption of microbial balance in the CDI group (0.68 ± 0.03 vs. 0.59 ± 0.02; *p* = 0.007). Pielou’s Evenness index further confirmed the homogenous distribution of microbial populations in the control group in comparison with the CDI group (0.68 ± 0.05 vs. 0.56 ± 0.04; *p* = 0.012) (Figure 7b).

(a) Examples of SEM micrographs obtained using the optimized protocol from samples of the Control and CDI groups. Low magnification (x100, stitched) shows the entire sample spot and thus the richness of the GM, while High magnification (x2,000) provides a detailed view of the microbial diversity within the GM. (b) α-diversity comparison of the gut microbiota between Control and CDI groups. Boxplots for each α-diversity index show a box representing the interquartile range (IQR) with the horizontal line indicating the median. Whiskers extend to the IQR range. Dots represent outliers beyond this range. *p-value <0.05. **p-value <0.01.

### Stool samples storage and stability over time using the optimized protocol

**Figure 8.**
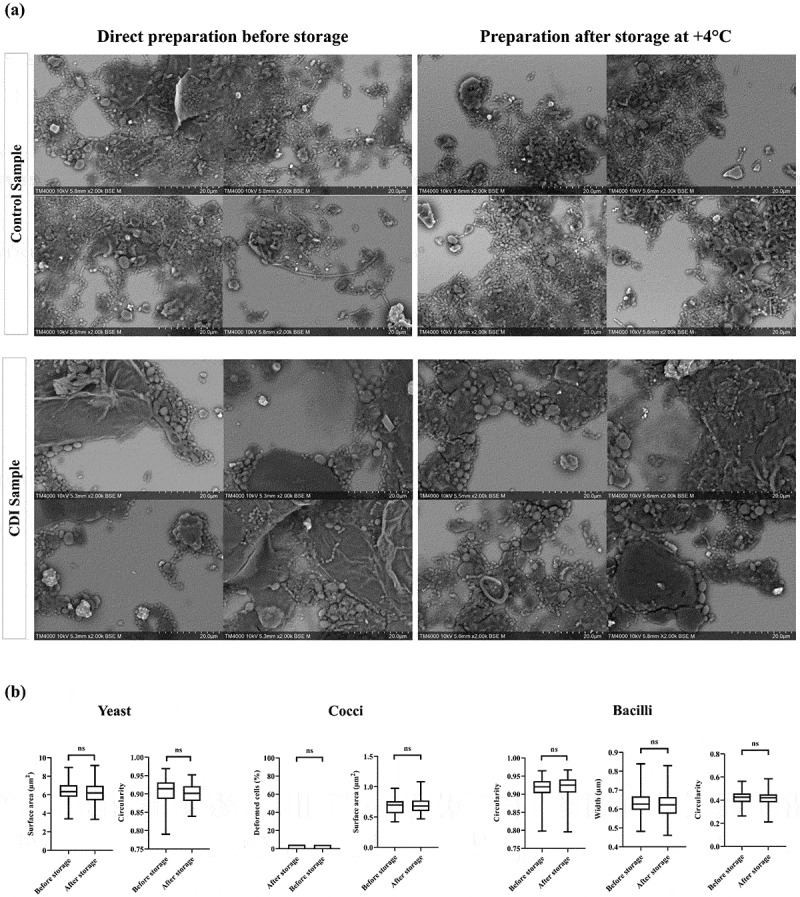
Stool preservation and stability after four months of storage at + 4°C.

Comparative analysis of SEM micrographs obtained before and after processed sample storage revealed a preserved overall sample integrity and no statistically significant difference in the morphology of GM components (Figure 8a). Yeasts preserved their oval or round shape with a smooth outer surface. Bacilli preserved their rod-shaped structure, low circularity and well-defined edges, while cocci maintained their spherical or ovoid forms without distortion (Figures 8b). Mega cells exhibited intact membranes and well-preserved surface details.

(a) SEM micrographs from randomly selected areas before and after storage. (b) Morphological characteristics of objects observed in SEM micrographs analyzed before and after four months of storage, including yeasts, cocci, and bacilli. Error bars represent the standard deviation (SD).

## Discussion

Despite multiple attempts for deciphering the complexities of the human gut microbiota, the ultimate method providing a complete description and landscape GM composition analysis remains elusive. Most existing methods are insufficient to provide a complete and reliable understanding of this complex ecosystem. This work presents a novel complementary approach for performing an in-depth exploration of GM components using a tabletop scanning electron microscope by means of a rapid and simplified sample preparation protocol. In this pioneering proof-of-concept, we demonstrate the feasibility in describing the difference in GM composition in the context of *C. difficile* infection, a well described dysbiosis-associated disease.

Initially, an optimized stool sample preparation protocol for SEM observation was developed. This protocol was selected for easy and rapid sample preparation, allowing SEM observation from sample to image in around 20 minutes. The protocol also ensured a homogenous stool sample deposition on the SEM-suitable support, which was crucial for an adequate and reproducible representation of the sample’s diversity and richness. Additionally, this protocol proved to be significantly faster and less labor-intensive compared to traditional protocols for SEM observation and compared to other existing methods for studying GM composition. Time-consuming culture-based methods that restrict observations to culturable organisms, such as the complex and resource-demanding culturomics approach, requires several days of sample processing and involves multiple culture conditions.^23^ The culturomics approach also faces major challenges in culturing and maintaining certain fastidious microorganisms, aside from the complete absence of detection of dead bacteria that are present in the sample.^29^ In fact, applying the novel SEM protocol will allow researchers to visualize the entirety of GM components within a sample, including culturable and non-culturable microorganisms, both live and dead, as well as human cells and debris. Likewise, this ability to directly visualize objects on SEM micrographs harbors a major advantage over methods that rely on molecular biology and genomics, as those methods provide a more obscure result with several probabilities that depend on sample quality and quantity.^30^ Specifically, metagenomic techniques have reduced sensitivity to minor bacterial populations and are affected by variability in DNA extraction or amplification kits between runs, potentially impacting outcomes.^30,31^ Therefore, the approach presented herein is expected to significantly enhance researchers’ ability to visualize the genuine components of GM, which can synergistically complement other more complex methods to help better describe the GM landscape.

At the development level, this proof-of-concept was built upon a novel tabletop SEM technology, which permits a rapid and easy to use SEM observation, unlike traditional SEMs. This makes the method significantly more accessible and provides immense flexibility in acquisition and preparation approaches, which in turn simplifies the optimization process. Most importantly, the power of this novel tabletop SEM technology lies in its ability to rapidly produce high-resolution micrographs at low vacuum, avoiding traditional SEM challenges like sample charging and possible image quality degradation upon repeat or prolonged acquisition times. This rapid acquisition allowed the establishment of a preliminary SEM database consisting of major GM components, based on the observed morphological features of different objects. This approach was similarly piloted in 2022 by Yimagou et *al*. through the creation of a repertoire of diverse objects within healthy microbiota using transmission electron microscopy (TEM), based on morphological traits as the basis for classification.^32^ However, that approach presented several challenges, including the lengthy sample preparation and variability in the distribution of bacterial cells in the ultrathin sections, which were heavily dependent on the preparation process. The current SEM-based method circumvents these limitations through the use of a rapid and simplified sample preparation method coupled with the novel tabletop SEM technology to provide a rather confident estimate of gut microbiota composition based on detected microbial morphologies. Protocol simplicity and microbiological representativeness were prioritized over rigid and complex standardization, which helped reveal realistic results and allowed for adequate randomization for reducing sampling bias while maintaining methodological reproducibility. Extensive studies have been made to standardize stool sample preparation for GM studies,^33,34^ however, it remains evident that standardizing stool samples at the level of sample preparation by weight or extensive homogenization can prove to be overly complex and introduce potential biases. Homogenizing stool samples by weight risks altering microbial representation, as some studies demonstrate that such methods selectively enrich or deplete specific taxa (e.g., increasing *Faecalibacterium* and *Bifidobacterium* while reducing Bacteroides and Parabacteroides).^34^ Standardizing stool aliquots by volume instead of weight also mitigates artificial biomass augmentation in less microbially dense samples, as fixed-volume sampling from heterogeneous stools intrinsically randomizes microenvironmental variations (ex. sample consistency) without amplifying technical biases linked to homogenization and weight-determination protocols. Nevertheless, weight-based comparisons may retain utility within homogenous sample groups (such as dysbiosis-related diseases), as these could help demonstrate and compare the lower microbial density within these groups.

To evaluate this proof-of-concept, a well-established dysbiosis-related disease model was chosen. SEM analysis of GM changes in the context of CDI revealed significant differences in GM composition, with distinct clustering observed between the CDI and control groups. This clustering was significant and correlated with the original distribution of the samples into the two analyzed groups, despite the small sample size (*n* = 10). Previous studies have demonstrated significant shifts in microbial populations including species alteration and depletion within the context of CDI and other dysbiosis-related diseases. This novel approach facilitated the observation of a depletion of both short bacilli and long bacilli in the CDI group, accompanied by a relative increase in yeasts. Effectively, a significant decrease in bacilli (short and long) was observed in the CDI group. These findings align with current knowledge on CDI pathophysiology and how antibiotic use disrupts GM,^35,36^ leading to the depletion of commensal bacilli, reducing colonization resistance, and allowing *Clostridioides difficile* to germinate and overgrow. In support of these findings, Amrane *et al*.^37^ also reported, using metagenomics and culturomics approaches, differences in microbial composition between control and CDI groups, including a depletion of *Bacteroidetes* and *Actinobacteria* (represented by bacilli) and an increase in the fungal fraction. SEM analysis also revealed a significant reduction in GM diversity and richness in the CDI group which was consistent with prior metagenomics and culturomics studies.^38–40^ Furthermore, the feasibility of detecting various human cells using SEM facilitated the observation of a significant increase in red blood cells and a relative increase in mega cells fractions in the CDI group compared to the control group. These findings were also in line with the known pathophysiology of CDI, where inflammation typically leads to increased mucus formation and bloody diarrhea in some cases.^41^

Overall, these findings underscore the potential of this approach in tracking GM dysbiosis and potential identification of specific markers of disease onset and progression. However, determining the potential of this methodology for future clinical applications still requires several improvements. This proof-of-concept was built upon the classification of detected objects that were defined based on well-described microorganism morphology, such as size, shape, and arrangement of bacteria, including length of bacilli and grouping patterns of cocci.^42^ This categorization was useful in interpreting the results for the analyzed dataset. However, as a larger number of samples are analyzed, including those representing other dysbiosis-related diseases and those from healthy individuals, the number of detected and classified objects is expected to expand, and additional layers of variables must be added to maximize the yield of information from the generated SEM images. For example, a set of objects that are exclusively related to a state of dysbiosis could be identified and tracked. Further investigations to improve the inherent value of this approach by the identification of SEM-based markers for determining bacterial viability status^43,44^ and bacterial identification are underway. This will further improve the readout of this approach and pave the way for a more accurate correlation with other available methods for studying microbial populations. Yet, this method offers direct access to the structural morphological diversity of the GM components, which can effectively complement existing methods and provide new insights into this complex microbial ecosystem as one of the tools comprising a multiomics approach for GM investigation.

On the other hand, the preservation of sample integrity over time is a powerful characteristic that allows the application of this method for comparative and follow-up studies, especially in tracking dysbiosis-related disease recurrence and therapeutic success. Future research aims at performing such comparative studies to encompass a more diverse range of samples from eubiotic and dysbiotic environments. Nevertheless, the promising nature of the results obtained with the current method and other works in progress provide an immense potential for such future applications.

To the best of our knowledge, this work is the first attempt to investigate morphological GM composition in the context of a dysbiosis-related disease using SEM. Further research comparing the GM composition of patients with symptomatic CDI versus asymptomatic carriers could provide valuable insights into the pathophysiology of this recurrent disease, a question that remains unresolved despite numerous metagenomics studies.^39^ Furthermore, these studies must include *C. difficile* strains with a variety of toxigenic profiles, spore formation dynamics, and resistance mechanisms. This approach could also be applied in a broader context, exploring other dysbiosis-related diseases and microbial ecosystems, such as the oral and vaginal microbiota.

Finally, it is evident that the current proof-of-concept evaluated a small subset of samples, and further studies are needed to demonstrate the reproducibility of this approach with larger cohorts of samples from diverse ecosystems and dysbiosis-related diseases. It is important to highlight that this proof-of-concept is dynamic and will continue to evolve as these additional samples are analyzed and as more objects are identified and integrated into future analyses. Furthermore, improvements must be applied to the current manual data analysis methodology, eventually automating this SEM analysis method by coupling automated image acquisition with real-time object detection and analysis by means of advanced machine learning models. At that point, this proof-of-concept could be transformed into a stronger GM landscape analysis tool, with promising clinical utility following the definition of biomarkers suitable for tracking early disease development, progression, or recurrence after treatment.

## Supplementary Material

Supplemental Material

## Data Availability

All data generated or analyzed during this study are included in this published article and its supplementary information files. All original data used in this article is available upon request.
