# Supplementary material for: Tips and tricks for gut microbiota investigation using scanning electron microscopy (SEM): going from sample preparation to imaging and landscape analysis
Source: Gut Microbes. 2025 Jun 9;17(1):2512016. doi: 10.1080/19490976.2025.2512016 (PMC12153398; doi:10.1080/19490976.2025.2512016)

**Supplementary Figure**

**Supplementary Figure 1.** General description of the method for measurement of object morphological characteristics on SEM micrographs using ImageJ Fiji software**.**


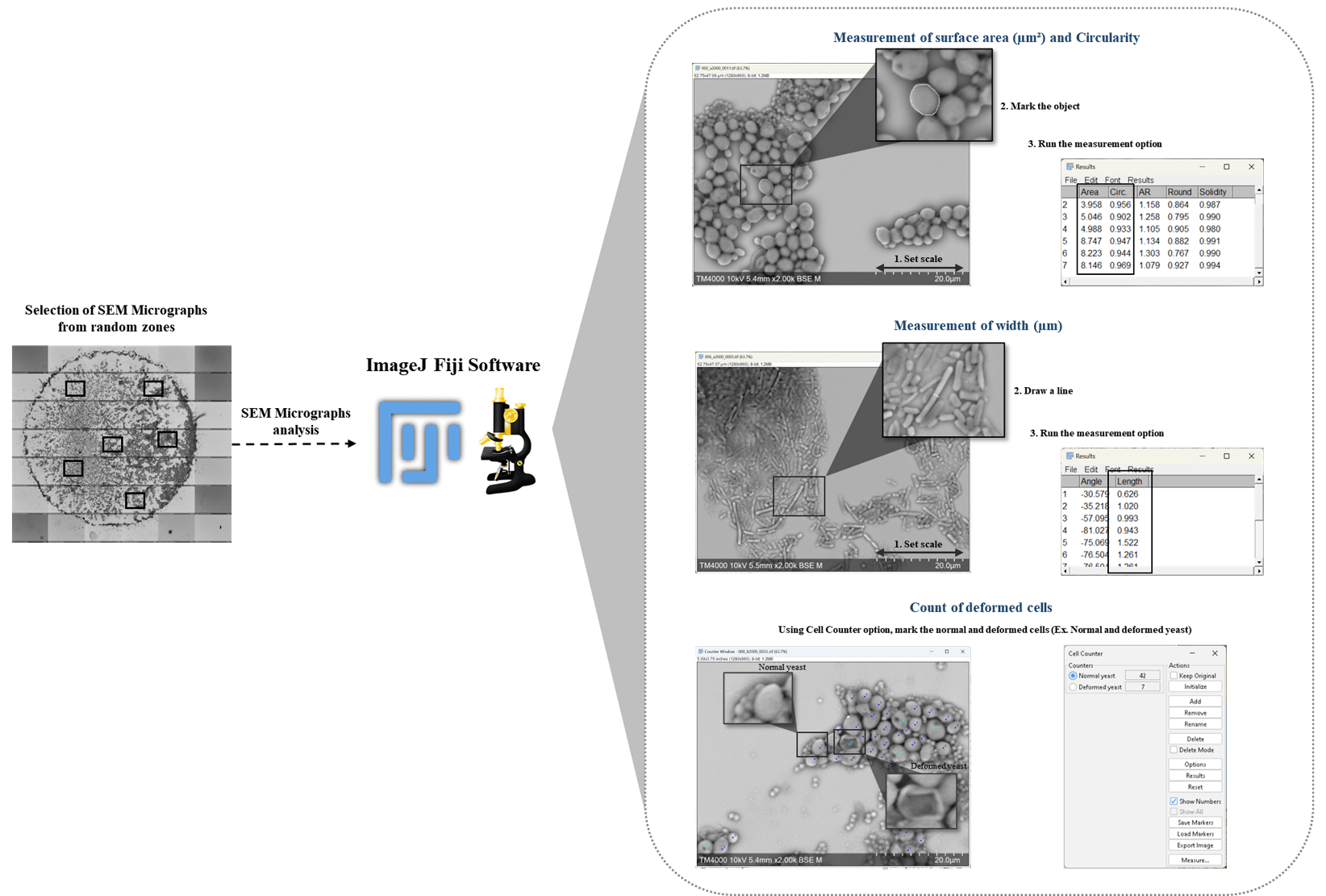

Supplement: Supplemental Material [file KGMI_A_2512016_SM8594.docx]
